# The cardiac work-loop technique: An *in vitro* model for identifying and profiling drug-induced changes in inotropy using rat papillary muscles

**DOI:** 10.1038/s41598-020-58935-2

**Published:** 2020-03-24

**Authors:** Sophie Fletcher, Helen Maddock, Rob S. James, Rob Wallis, Mayel Gharanei

**Affiliations:** 1 Centre for Sport, Exercise and Life Sciences, Coventry University, Coventry, United Kingdom; 2 InoCardia Ltd, Technocentre, Puma Way, Coventry, CV1 2TT UK

**Keywords:** Drug safety, Pharmaceutics, Pharmacology, Cardiovascular biology

## Abstract

The cardiac work-loop technique closely mimics the intrinsic *in vivo* movement and characteristics of cardiac muscle function. In this study, six known inotropes were profiled using the work-loop technique to evaluate the potential of this method to predict inotropy. Papillary muscles from male Sprague-Dawley rats were mounted onto an organ bath perfused with Krebs-Henseleit buffer. Following optimisation, work-loop contractions were performed that included an initial stabilisation period followed by vehicle control or drug administration. Six known inotropes were tested: digoxin, dobutamine, isoprenaline, flecainide, verapamil and atenolol. Muscle performance was evaluated by calculating power output during work-loop contraction. Digoxin, dobutamine and isoprenaline caused a significant increase in power output of muscles when compared to vehicle control. Flecainide, verapamil and atenolol significantly reduced power output of muscles. These changes in power output were reflected in alterations in work loop shapes. This is the first study in which changes in work-loop shape detailing for example the activation, shortening or passive re-lengthening have been linked to the mechanism of action of a compound. This study has demonstrated that the work-loop technique can provide an important novel method with which to assess detailed mechanisms of drug-induced effects on cardiac muscle contractility.

## Introduction

The primary function of the heart is as a mechanical pump; undergoing two main phases during each cardiac cycle: contraction (systole) and relaxation (diastole). A change in contractility, as a result of pharmacological interventions (inotropy) or a pathophysiological condition, would therefore impact on the hearts intrinsic ability to act as a mechanical pump. Hence, it is important to have a reliable index of contractility both *in vivo* and *in vitro*.

The most reliable way to measure changes in contractility *in vivo* is by measuring changes in left ventricular pressure and volume, as first suggested by Suga and Sagawa^1^. The relationship between pressure and volume has since become a widely accepted index of cardiac contractility^2^. Because of the general applicability of this concept to hearts of all species, pressure-volume analysis has become the favoured practice for assessing contractility in pre-clinical studies of all mammals including mice, dogs and non-human primates. Telemetry studies involving conscious animals are often considered to be the gold standard for pre-clinical contractility assessment; often by measuring changes in pressure or volume or other measures such as LVDP/dt max^3^. However, the use of telemetry is expensive, has a relatively slow throughput, and requires the use of live animals. Hence, such measurements are often carried out late in the development of new pharmacological compounds.

Although the pressure-volume relationship is the most direct and reliable way to measure contractility *in vivo*, in a clinical setting the pressure-volume relationship is rarely measured directly to assess changes in contraction and heart function. The use of conductance catheters to measure this relationship is highly reliable, however, it is invasive, complex, expensive and technically demanding^2^. One technique routinely used to identify changes in cardiac performance and contractility in a clinical setting is the method of echocardiography; it is currently recommended by National Institute for Health and Care Excellence in the diagnosis and monitoring of acute and chronic heart failure^4,5^. Echocardiograms are commonly used to assess the structure and function of the heart. Function is quantified using estimations of cardiac output, ejection fraction and diastolic function. The biggest advantages of echocardiography are that it is non-invasive and relatively inexpensive meaning it can be used routinely. However, haemodynamic parameters such as cardiac output and ejection fraction are not measured directly but estimated based on other measurements^6,7^, meaning there is a higher degree of error compared to more direct measurements.

It is important to have a reliable *in vitro* measure of cardiac contractility which can be used in a translative manner in order to predict changes *in vivo* and to help us better understand complex pathophysiological conditions. There are many *in vitro* techniques which can be used to assess contractility, ranging from whole heart to those using single cardiomyocytes. A well-established *in vitro* technique commonly used to assess changes in contractility is the Langendorff perfused heart model^8^. The Langendorff technique utilises the whole heart (routinely small mammals such as rats or mice) which is perfused in a retrograde manner under constant pressure. One of the main benefits of this technique is that there are no diffusion constraints as the vasculature of the heart is utilised in the perfusion of compounds it is also possible to acquire electrocardiogram traces during experiments^8^. One of the main disadvantages is that there is a lack of afterload which does not reflect biomechanics *in vivo*.

Muscles isolated from the myocardium can also be utilised *in vitro* and are predominantly used in isometric contraction studies, during which the muscles are held at a constant length and stimulated to produce a twitch response^9,10^. However, *in vivo* cardiac muscle is rarely at constant length during contraction and relaxation, hence, isometric contraction studies do not fully replicate the dynamic cardiac muscle movement observed *in vivo*. Isometric contraction studies also overestimate the power output of a muscle as they do not account for the fact that muscles cannot be continuously maximally activated; time is required for relaxation, re-lengthening and activation^11–14^.

Another class of techniques currently used experimentally to assess contractility are cellular assays such as impedance- or sarcomere shortening- assays^15–18^. These techniques have the potential to be higher throughput than those mentioned previously. However, one main drawback of these methods is that generally the cardiomyocytes are unloaded which is not representative of *in vivo* conditions. Also, impedance is not a direct measure of contraction but merely a surrogate.

In isolated muscles or cells, it is not possible to measure changes in pressure and volume because the atria and ventricles are no longer intact. In the cardiac work-loop technique changes in force and length are measured instead. The technique incorporates a sinusoidal length change along with electrical stimulation that closely mimics the cardiac cycle *in vivo* and has the potential to be used pre-clinically to identify changes in contractility. It has been suggested that strain patterns that occur *in vivo* are almost sinusoidal in shape^19,20^, whilst the exact strain patterns are more complex in nature, a sinusoidal length change offers a good approximation of *in vivo* muscle mechanics^21^.

The work-loop technique has previously been utilised in studies of both skeletal^11,22,23^ and cardiac muscle^12,24–29^. Layland *et al*.^28^ have previously investigated the effect of 5 µM isoprenaline and 100 µM of phenylephrine on the power output of isolated rat ventricular trabeculae muscles. This study successfully demonstrated that both isoprenaline and phenylephrine increased the power output of the trabeculae muscles using the cardiac work-loop technique and hence were positive inotropes. However, the study was not performed at a physiologically relevant temperature (24 °C) and relatively high concentrations of both compounds were used.

Prior to the current study, extensive work has been carried out in our laboratory in order optimise the cardiac work-loop technique using rat papillary muscles under physiologically relevant conditions (temperature, length change and cycle frequency) (data not published).

In the current study, isolated rat papillary muscles were used to assess the ability of the cardiac work-loop technique to identify drug-induced changes in contractility by testing six known inotropes. The power output of the muscles was measured and compared statistically to a suitable vehicle control. The shape of the work-loop during drug treatment was compared to that during stabilisation and any changes in the work-loop shape were linked to the mechanism of action of the compound. The aim of the study was to demonstrate that the cardiac work-loop using rat papillary muscles has the potential to be used as an *in vitro* technique to identify drug induced changes in contractility.

## Materials and Methods

### Chemicals

Isoprenaline hydrochloride, atenolol and verapamil hydrochloride were purchased from Abcam (Cambridge, UK). Dobutamine hydrochloride, digoxin and flecainide acetate were purchased from Tocris Bioscience (Oxford, UK). Stock solutions (1–10 mM) of all compounds were made and were stored at −20 °C. Digoxin and dobutamine were dissolved in dimethyl sulfoxide (DMSO) to make the stock solution. The final concentration of DMSO during experimentation was less than 0.01%. Verapamil, atenolol, flecainide and isoprenaline were all dissolved in ultra-pure water to make the stock solution. All other reagents were purchased from either Sigma Aldrich (Dorset, UK) or Fisher Scientific (Loughborough, UK) unless stated otherwise.

### Animals

Male Sprague-Dawley rats were used in all experiments; they were obtained from Charles River (Margate, UK) and housed at the University of Warwick. Experiments were conducted in accordance with the Guidelines on the Operation of Animals (Scientific Procedures Act 1986) and were approved by the Coventry University ethics committee.

### Papillary muscle dissection

Muscle preparation was similar to that previously described^12^. Rats were sacrificed via cervical dislocation; the hearts were then carefully and rapidly excised and placed into ice-cold, oxygenated (100% O_2_) Krebs Henseleit (KH) buffer. The KH buffer comprised of: NaCl (144 mM), Na Pyruvate (10 mM), HEPES [4-(2-hydroxyethyl)−1-piperazineethanesulfonic acid] (10 mM), KCl (6 mM), CaCl_2_·2H_2_O (2 mM), MgCl_2_·6H_2_O (1 mM), NaH_2_PO_4_ (1 mM) and MgSO_4_·7H_2_O (1 mM). The buffer was oxygenated and heated to 37 °C for 30 minutes. The pH was adjusted to 7.40 using 5 M NaOH prior to its use.

Dissections were carried out under a dissecting stereomicroscope using a petri-dish containing Sylgard 184 Silicone Elastomer Polymer (Farnell, UK). Throughout the dissections, the tissue was submerged in cold (2 °C–4 °C), oxygenated KH buffer which was regularly discarded and replaced. The heart was carefully pinned to the dish then a small incision was made at the apex using microfine scissors. Cuts were made carefully along the wall of the left ventricle and along the intraventricular septum exposing the papillary muscles. Papillary muscles were then selected based on size and shape; a T-clip (a small T-shape made of tin foil) was attached to either end of the chosen muscle. Following dissection, the muscles were placed onto a work-loop rig consisting of: a horizontal organ bath (which included platinum stimulating electrodes and a micromanipulator), high-speed length controller, 100 mN/50 mN capacity force transducer, stimulator, thermocouple and signal interface (all from Aurora Scientific, Canada). The organ bath was perfused with recirculating KH buffer, which was gassed continuously. The temperature within the organ bath was maintained at 37 °C throughout the experiments. The muscles rested under a passive force of ~0 mN for at least 20 minutes before length optimisation.

### Muscle length optimisation

Once the muscles had acclimatised to 37 °C, the muscles were slowly stretched using a micromanipulator resulting in a small amount of passive force (around 3 mN), they were then stimulated while kept at a constant length (isometric). The stimulation produced an isometric twitch and the developed force was calculated by bespoke software by subtracting the minimum force (passive force) from the maximum force (active force). The software used had been developed in-house; it was used for length optimisation and work-loop protocols it was able to display real-time work-loop data during work-loop protocols.

The length was increased incrementally, and a stimulus delivered at each length, until a muscle length at which maximum developed force was reached (L_max_). The length of the muscle was measured using a microscope fitted with an eyepiece graticule and was then adjusted to 90% of L_max_ (in accordance with preliminary optimisation experiments, data not shown here).

### Work-loop protocol

**Figure 1:**
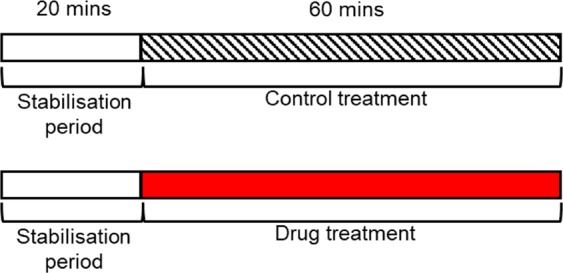
Depiction of protocols used. Work-loops were carried out every 5 minutes during the protocols.

Figure 1 shows the two protocols used, during both protocols, work-loop contractions were carried out every 5 minutes for 80 minutes. As detailed, the first 20 minutes was used as a stabilisation period to determine baseline muscle performance. Preparations that were unstable during the stabilisation period (greater than 20% variation in power output from the mean value during stabilisation) were excluded from the data set. Following the stabilisation period, muscles in the vehicle control groups were perfused with either KH buffer or 0.01% DMSO solution (dissolved in KH buffer). A wash-out was not included in this protocol in order to increase the throughput of the technique.

During all experiments, work-loop contractions were carried out using the following parameters: initial length of 90% L_max_, strain amplitude =  ± 4%, cycle frequency = 6 Hz, pulse width of stimulation = 1.0 ms and a current of 120 mA was used for all stimulations (parameters were established during preliminary optimisation experiments, data not shown).

At the end of the experiments, the wet muscle mass was measured to the nearest 0.00001 g using an electronic balance (Sartorius BP211D, Germany). Muscle mass, fibre length and the assumed muscle density of 1060 kg m^−3^ ^12,30^ were used to calculate fibre cross-sectional area and the isometric muscle stress.

### Analysis

**Figure 2:**
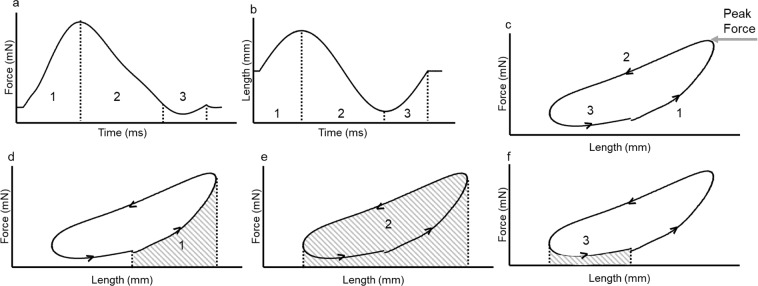
Adapted from Gharanei *et al*. 2014 showing: (**a**) typical force, (**b**) length and (**c**–**f**) work-loop traces. The cardiac work-loop consists of three main phases which have been labelled in each diagram. (**a**) typical force trace of a cardiac work-loop. (**b**) typical sinusoidal length trace of a cardiac work-loop. (**c**) typical work-loop trace showing 3 main phases: (1) Activation of the muscle. (2) Shortening of the muscle (3) Passive re-lengthening of the muscle. (**d**) Phase 1 is highlighted- during this phase the muscle is simultaneously stimulated electrically and lengthened. The shaded area depicts the work required to lengthen the muscle from initial length to maximum length. (**e**) Phase 2 is highlighted- once the peak force has been reached the length of the muscle is shortened to below that of the original length. The force gradually decreases during this period. The area highlighted during this phase is the total work done during shortening. (**f**) Phase 3 is highlighted- during this phase the muscle is re-lengthened back to its starting length. The area highlighted is the work required to re-lengthen the muscle to initial length. The net work, which corresponds to the area within the loop can be calculated as: area 2 – (area 1 + area 3).

Work-loop traces were produced by plotting the force against the length (or strain) of the muscle. Figure 2 depicts a typical cardiac work-loop and its three main phases. It also depicts how net work can be calculated based on the net area of the loop. In this study net power output was calculated which is proportional to the net work. Instantaneous power was calculated for each data point (1667 points per loop) by multiplying the instantaneous force by the instantaneous velocity^12^. The instantaneous power output values were then averaged to give average net power output value. The average net power output value was then adjusted to account for muscle mass. For each of the time courses, the power output during the vehicle- or drug- treatment was expressed as a percentage of the average power output during stabilisation.

All six drug treatments were compared to a time-matched vehicle control treatment. The data were expressed as mean ± SEM. Power output values were assessed for statistical differences, *p* < 0.05 was considered statistically significant. A repeated measure two-way analysis of variance (ANOVA) was performed, if the ANOVA was significant (p < 0.05) independent sample t-tests were performed for each time point. The two vehicle control groups (KH buffer and 0.01% DMSO) were also compared to each other using a two-way repeated measure ANOVA, there was no significant difference in the power output (p = 0.104).

During the initial nine dobutamine experiments four experiments had to be excluded from the analysis and repeated. In these cases, dobutamine treatment caused the muscles to contract spontaneously causing the muscle to fatigue, resulting in work-loops which did not represent the true power output of the muscle.

## Results

### Muscle characteristics

The characteristics of the muscle preparations following length optimisation and before any drug or control treatment are shown in Table 1. All experimental groups were compared using a one- way ANOVA (analysis of variance) with post-hoc Bonferroni test. There were no significant differences (p > 0.05) between the experimental groups for the following characteristics: L_max_, active force, passive force, developed force, fibre CSA and stress (all at L_max_), muscle mass and net power output at the end of stabilisation.

**Table 1:** Characteristics of papillary muscle preparations.

	KH Vehicle Control (n = 7)	0.01% DMSO Vehicle Control (n = 7)	Digoxin (n = 6)	Dobutamine (n = 9)	Isoprenaline (n = 7)	Verapamil (n = 7)	Flecainide (n = 7)	Atenolol (n = 8)
**L**_**max**_ **(mm)**	2.10 ± 0.71	2.71 ± 0.72	1.85 ± 0.57	2.19 ± 0.41	1.70 ± 0.62	2.18 ± 0.52	2.20 ± 0.87	2.45 ± 0.45
**Active Force (mN)**	17.4 ± 4.5	23.2 ± 6.5	17.3 ±3.8	23.1 ± 7.4	19.9 ± 4.3	21.5 ± 8.3	24.4 ± 10.8	20.7 ± 7.5
**Passive Force (mN)**	5.4 ± 1.4	6.2 ± 2.1	6.1 ± 1.5	7.5 ± 1.3	7.1 ± 1.4	5.8 ± 1.6	8.2 ± 1.3	5.8 ± 2.1
**Developed Force (mN)**	11.9 ± 3.5	17.0 ± 5.3	11.2 ± 3.1	15.6 ± 6.2	12.8 ± 3.1	15.7 ± 7.6	15.5 ± 10.1	14.8 ± 6.4
**Fibre CSA (m**^**2**^**)**	3.2 × 10^−7^ ± 1.2 × 10^−7^	2.7 × 10^−7^ ± 1.0 × 10^−7^	4.3 × 10^−7^ ± 2.6 × 10 ^−7^	2.7 × 10^−7^ ± 1.1 × 10^−7^	2.5 × 10^−7^ ± 1.4 × 10^−7^	4.1 × 10^−7^ ± 1.2 × 10^−7^	2.7 × 10^−7^ ± 1.1 × 10^−7^	3.0 × 10^−7^ ± 1.4 × 10^−7^
**Isometric Stress (kNm**^**−2**^**)**	41 ± 16	65 ± 10	37 ± 23	60 ± 15	56 ± 21	41 ± 25	49 ± 19	48 ± 16
**Muscle Mass (µg)**	677 ± 282	772 ± 304	778 ± 397	636 ± 305	554 ± 452	866 ± 392	684 ± 481	873 ± 431
**Average net power (end of stabilisation) (Wkg**^**−1**^**) (at 90% L**_**max**_**)**	9.9 ± 4.7	18.2 ± 3.5	10.1 ± 6.7	15.1 ± 4.0	14.8 ± 8.1	9.7 ± 4.4	16.4 ± 7.8	14.2 ± 4.8

L_max_, length at which maximum force was produced; CSA, cross-sectional area. Unless specified all values are for when the muscle is at length L_max_ Values are mean ± SD (*n* = 6–9).

### Drug induced changes on muscle performance

**Figure 3:**
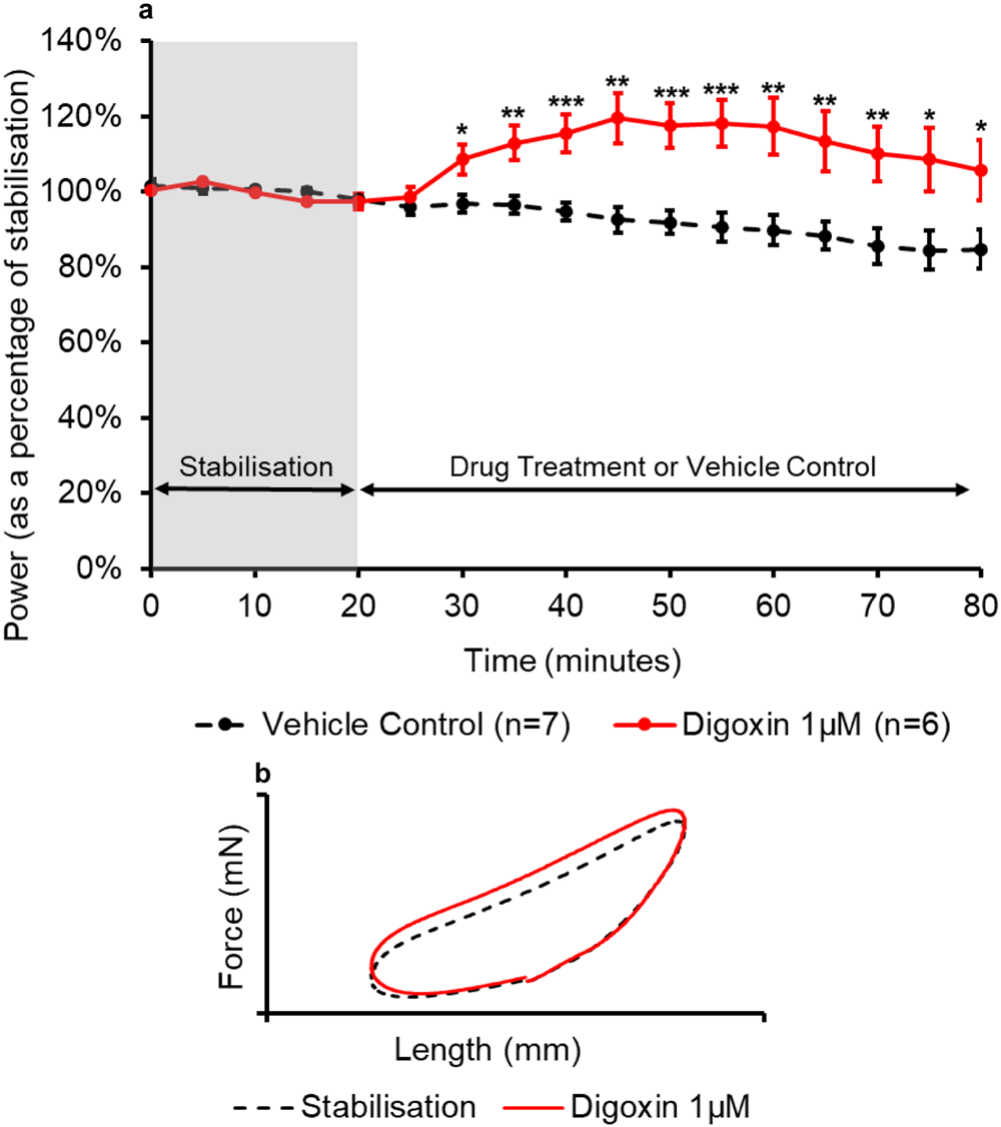
(**a**) The effect of digoxin administration on the power output of the muscle (n = 6) compared to the vehicle control treatment (n = 7). All muscles underwent a 20-minute stabilisation period (shown in grey); followed by 60 minutes of either control- or drug- treatment. The power values for the control- and drug- treatment are expressed as a percentage of the average value during stabilisation. Values are presented as mean ± SEM. (n = 6–7). Two-way repeated measures ANOVA followed by independent samples t-test for each time point DMSO vehicle control vs Digoxin: *p<0.05, **p<0.01 and ***p<0.001. (**b**) The effect of digoxin on representative work-loop shapes. The black dashed loop is from the end of the stabilisation period (20 minutes), the red loop is from the point of maximal drug response (55 minutes overall).

**Figure 4:**
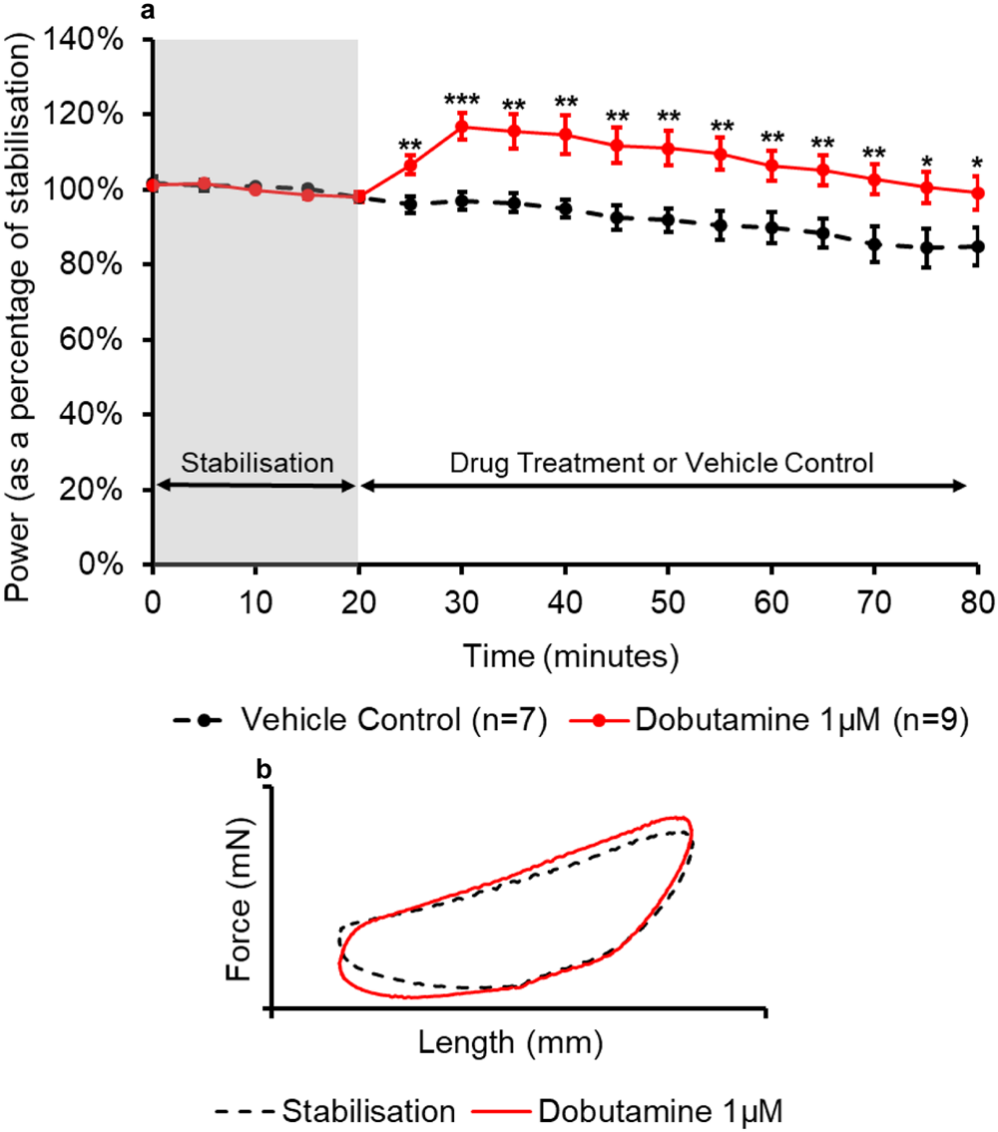
(**a**) The effect of dobutamine administration on the power output of the muscle (n = 9) compared to the control treatment (n = 7). All muscles underwent a 20-minute stabilisation period (shown in grey); followed by 60 minutes of either control- or drug- treatment. The power values for the control- and drug- treatment are expressed as a percentage of the average value during stabilisation. Values are presented as mean ± SEM. (n = 7–9). Two-way repeated measures ANOVA followed by independent samples t-test for each time point DMSO vehicle control vs Dobutamine: *p<0.05, **p<0.01 and ***p<0.001. (**b**) The effect of dobutamine on representative work-loop shapes. The black dashed loop is from the end of the stabilisation period (20 minutes), the red loop is from the point of maximal drug response (30 minutes overall).

**Figure 5:**
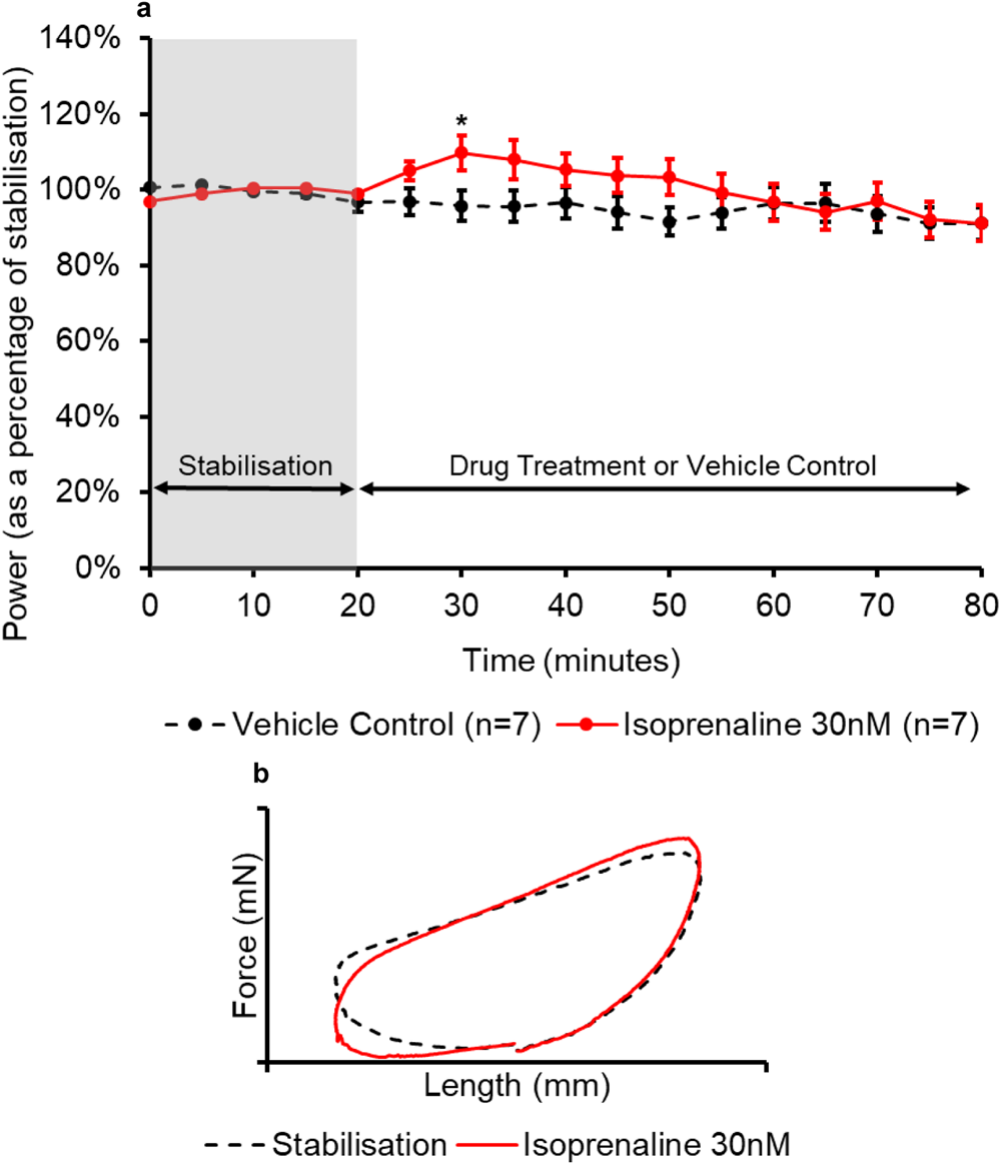
(**a**) The effect of isoprenaline administration on the power output of the muscle (n = 7) compared to the vehicle control treatment. All muscles underwent a 20-minute stabilisation period (shown in grey); followed by 60 minutes of either vehicle- or drug- treatment. The power values for the control- and drug- treatment are expressed as a percentage of the average value during stabilisation. Values are presented as mean ± SEM. (n = 7). Two-way repeated measures ANOVA followed by independent samples t-test for each time point KH vehicle control vs Isoprenaline: *p<0.05. (**b**) The effect of isoprenaline on representative work-loop shapes. The black dashed loop is from the end of the stabilisation period (20 minutes), the red loop is from the point of maximal drug response (30 minutes overall).

**Figure 6:**
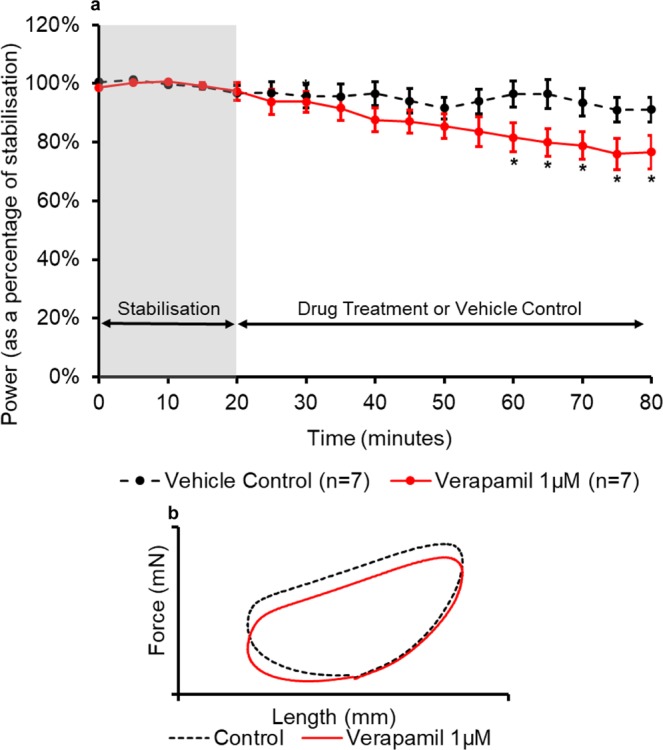
(**a**) The effect of verapamil administration on the power output of the muscle (n = 7) compared to the control treatment. All muscles underwent a 20-minute stabilisation period (shown in grey); followed by 60 minutes of either control- or drug- treatment. The power values for the control- and drug- treatment are expressed as a percentage of the average value during stabilisation. Values are presented as mean ± SEM. (n = 7). Two-way repeated measures ANOVA followed by independent Two-way repeated measures ANOVA followed by independent samples t-test for each time point comparing Verapamil and KH Vehicle Control: *p<0.05. (**b**) The effect of verapamil on representative work-loop shapes. The black dashed loop is from the end of the stabilisation period (20 minutes), the red loop is from the point of maximal drug response (65 minutes overall).

**Figure 7:**
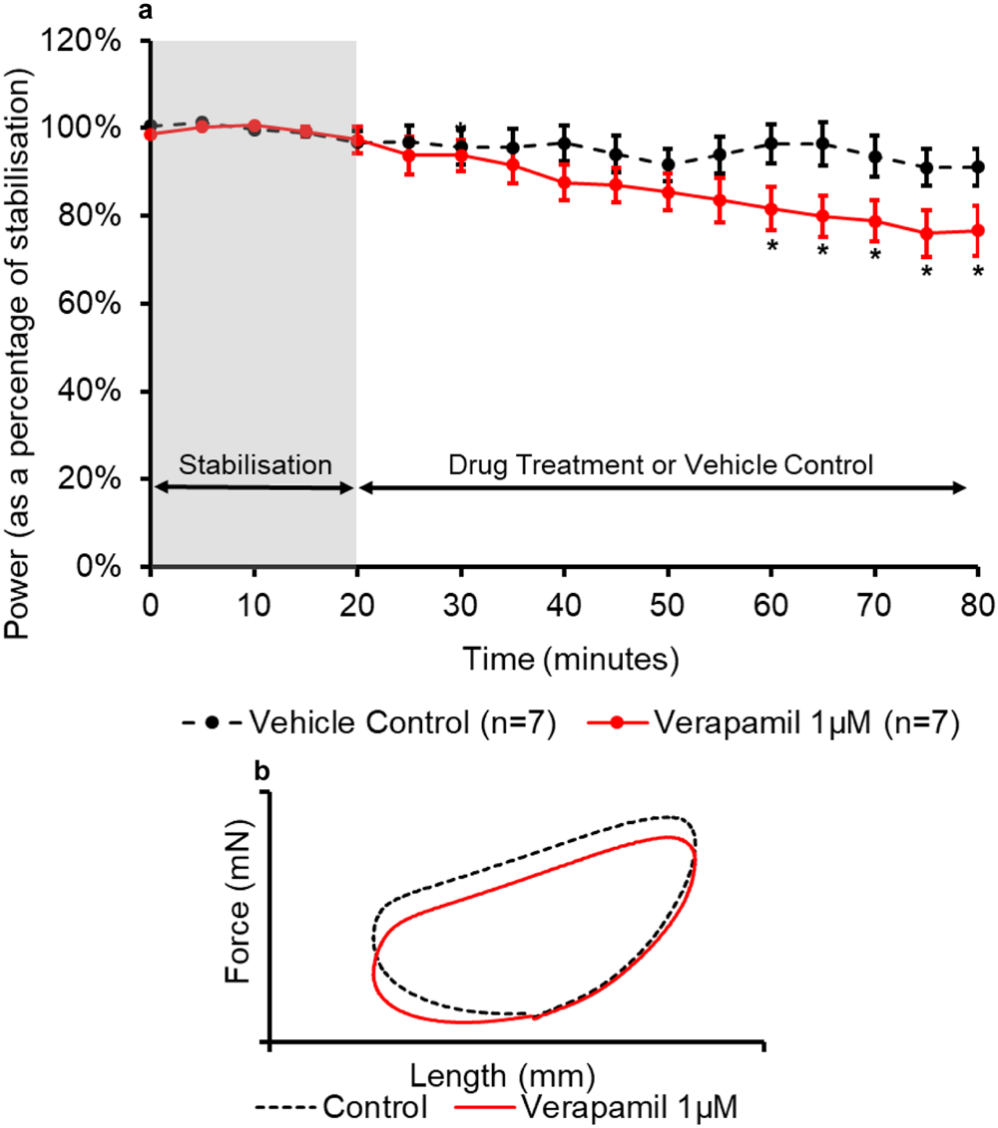
(**a**) The effect of flecainide administration on the power output of the muscle (n = 7) compared to the control treatment. All muscles underwent a 20-minute stabilisation period (shown in grey); followed by 60 minutes of either control- or drug- treatment. The power values for the control- and drug- treatment are expressed as a percentage of the average value during stabilisation. Values are presented as mean ± SEM. (n = 7). Two-way repeated measures ANOVA followed by independent samples t-test for each time point comparing Flecainide and KH Vehicle Control: *p<0.05 and **p<0.01. (**b**) The effect of flecainide on representative work-loop shapes. The black dashed loop is from the end of the stabilisation period (20 minutes), the red loop is from the point of maximal drug response (60 minutes overall).

**Figure 8:**
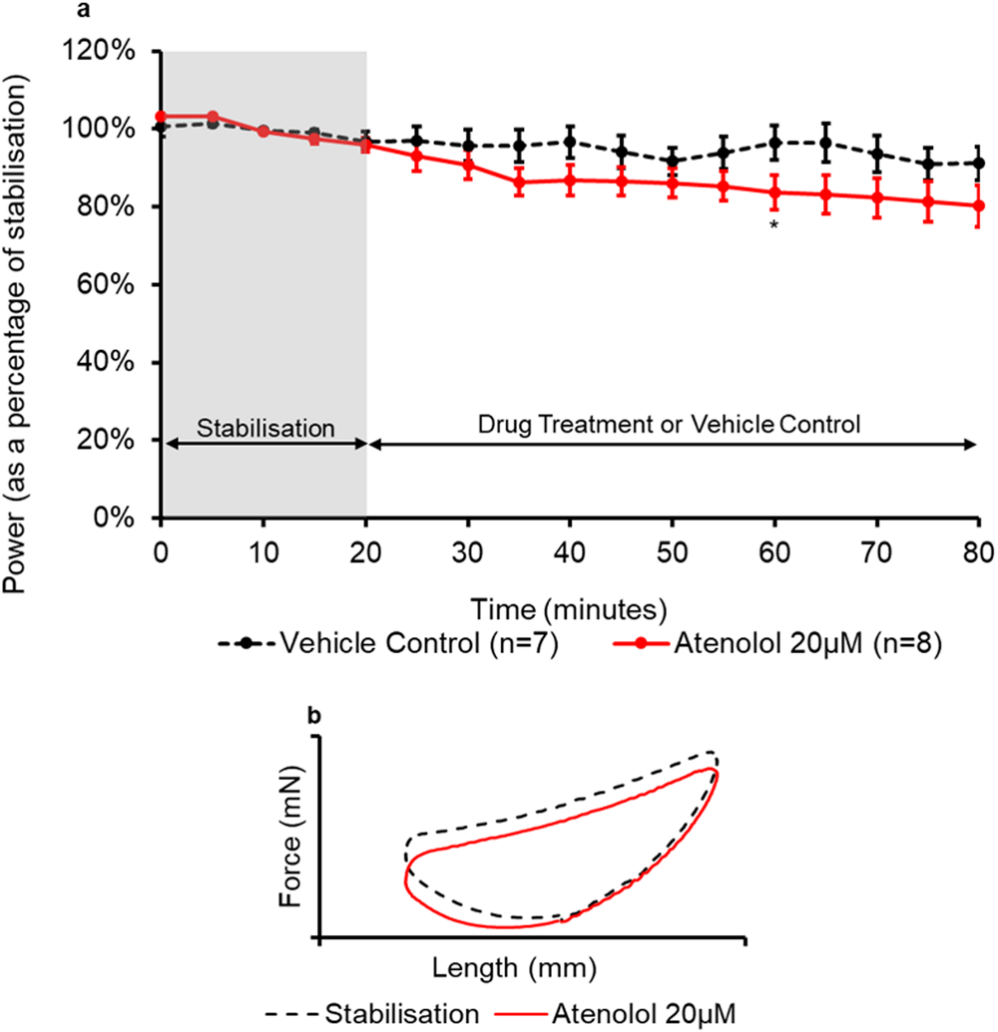
(**a**) The effect of atenolol administration on the power output of the muscle (n = 7) compared to the control treatment. All muscles underwent a 20-minute stabilisation period (shown in grey); followed by 60 minutes of either control- or drug- treatment. The power values for the control- and drug- treatment are expressed as a percentage of the average value during stabilisation. Values are presented as mean ± SEM. (n = 7–8). Two-way repeated measures ANOVA followed by independent samples t-test for each time point KH vehicle control vs Atenolol: *p<0.05. (**b**) The effect of atenolol on representative work-loop shapes. The black dashed loop is from the end of the stabilisation period (20 minutes), the red loop is from the point of maximal drug response (60 minutes overall).

For all six drug treatments, a time-course and a pair of representative work-loops have been presented in the figures below (Figs. 3, 4, 5, 6, 7, 8). The time-course depicts the mean ( ± SEM) power output of the muscle preparations over 80 minutes. The effect of positive and negative inotropes on the power output of the muscle over time are demonstrated and how they compare to the time-matched vehicle treatment. For the representative work-loops a loop from the end of stabilisation (20 minutes) has been compared to a work-loop from the time point of maximal drug response (based on power output values).

### Positive inotropes

As shown in Fig. 3a, digoxin (1 µM) caused a significant and sustained increase in the power output of the muscle. After 10 minutes of treatment, digoxin caused a significant (p = 0.033) increase in power output when compared to the time-matched vehicle control; a significant difference was maintained for the remainder of the experiment. The greatest increase in contractility was observed 30 minutes into the drug treatment with an increase in power output of 27% ( ± 9%) compared to the vehicle control treatment (p = 0.001). Digoxin elicited its effect primarily during the second phase of the work-loop where it caused an increase in the force throughout muscle shortening, there was also an increase in the peak force when compared to the work-loop from stabilisation (Fig. 3b).

Dobutamine (1 µM) also caused a significant increase in the power output of the muscle (Fig. 4a). After 5 minutes of drug treatment, dobutamine caused a significant increase in the power output of the muscle (p = 0.005) when compared to time-matched controls. The maximal response was recorded 10 minutes into the drug treatment with an increase in power output of 20% ( ± 6%) when compared to the vehicle control treatment. A significant increase in power output was sustained for the remainder of the experiment. Dobutamine elicited its effects during the active phase of the loop and the passive re-lengthening of the muscle (Fig. 4b). An increase in the peak force was observed, however, this increase in force was not sustained throughout the whole of the second phase. During the third phase, the force during re-lengthening was lower compared to stabilisation which reduced the negative work during re-lengthening to initial length and hence net power output.

Isoprenaline (30 nM) caused a significant increase in power output of 14% ( ± 9%) after 10 minutes of drug treatment when compared to the control (p = 0.03) (Fig. 5a). During the remainder of the experiment, the drug induced increase in muscle performance declined and reached power values comparable to the vehicle values. Isoprenaline also elicited its effects during the second and third phase of the work-loop in a similar manner to dobutamine causing an increase in peak work-loop force and a lower force during re-lengthening to initial length Fig. 5b.

### Negative inotropes

Verapamil (1 µM) caused a significant (p = 0.05) decrease in the power output of the muscle after 40 minutes of drug treatment (60 minutes overall) when compared to the time-matched control (Fig. 6a). The largest difference in power output was observed 45 minutes into the drug treatment, the power output was 17% ( ± 9%) lower compared to the time-matched vehicle control treatment. A significant reduction in power output was sustained for the remainder of the time course. Verapamil elicited its effects during the second and third phase of the work-loop causing a reduction in the force produced by the muscle during both phases including a reduction in the peak force when compared to stabilisation (Fig. 6b).

Flecainide 3 µM caused a significant (p = 0.009) decrease of 16% ( ± 6%) in the power output after 40 minutes of drug treatment when compared to the control (Fig. 7a). This decrease in power output remained significant until the end of the drug treatment (p < 0.05). Figure 7b shows that flecainide caused a reduction in the force produced by the muscle throughout all three phases of the work-loop including a reduction in the peak force at the beginning of phase 2.

Atenolol 20 µM caused a significant (p = 0.046) reduction of 13% ( ± 7%) in the power output of the muscle after 45 minutes of drug treatment (Fig. 8a). Figure 8b shows that atenolol caused a reduction in the force produced by the muscle during the second and third phase of the work-loop.

## Discussion

The heart is a dynamic organ and cardiac contraction is made possible by a complex interplay between the electrical and structural changes that directly result in the specific function of the heart^31^. The process is complex and specific meaning a small change in the structural, electrical or other changes can result in significant changes in the performance of the heart and its function. It is therefore imperative to regard the structural biomechanics of the heart when designing *in vitro* contractility assays.

**Figure 9:**
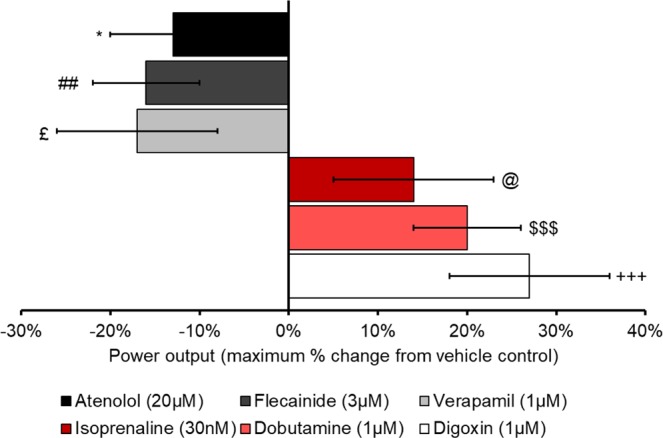
Maximal drug response for each compound when compared to appropriate vehicle control, results presented as mean ± SEM. *p = 0.046 atenolol vs KH vehicle control (60 minutes overall), ##p = 0.009 flecainide vs KH vehicle control (60 minutes), £p=0.041 verapamil vs KH control (75 minutes), @p=0.03 isoprenaline vs KH control (30 minutes), $$$p = 0.0004 dobutamine vs 0.01% DMSO control (30 minutes), +++p = 0.001 digoxin vs 0.01% DMSO control (55 minutes).

In this study, six known inotropes have been used to assess the suitability of the cardiac work-loop technique for identification of inotropes. Each of the compounds tested caused a significant change in the power output of the muscle when compared to the appropriate vehicle control. Digoxin, dobutamine and isoprenaline caused significant increases in the power output (Fig. 9) of the muscle and were therefore successfully classified as positive inotropes. Verapamil, flecainide and atenolol caused a significant decrease in the power output (Fig. 9) and were successfully classified as negative inotropes. The compounds used in this study were chosen because their activity *in vivo* and *in vitro* has been well documented within the literature. Table 2 details how the concentrations used in this study compare to other *in vitro* studies and human *in vivo* studies.

**Table 2:** Comparison between the concentration of compounds used in this study and concentrations used in other *in vitro* studies and the estimated free concentration which is based on the measured plasma concentration.

Compound	Concentration used in this study (µM)	*In vitro* studies		Human *in vivo* studies		
		Solution concentration (µM)	Reference	Plasma concentration (ng/ml)	Estimated free concentration (µM)	Reference
Digoxin	1	30	^36^	1.32	0.002	^54^
		0.3, 1	^37^			
		5	^38^			
		0.03–10	^39^			
Dobutamine	1	0.1–10	^10^	200	0.5	^55^
Isoprenaline	0.03	5	^27^	0.5	0.002	^56^
		5	^28^			
Verapamil	1	0.001–10	^44^	40	0.01	^57^
Flecainide	3	12,24	^46^	296	0.4	^59^
		0.01–100	^58^			
Atenolol	20	0.01–30	^9^	550	0.2	^60^
		~9.4	^52^			

### Positive inotropes

Work-loop data presented here shows that digoxin (1 µM) significantly increased cardiac muscle performance and caused elevated forces during muscle shortening as observed from the work-loop shape (Fig. 3). Digoxin is a cardiac glycoside and inhibits sodium-potassium ATPase (Na^+^/K^+^ ATPase)^32^. Inhibition causes an increase in the levels of intracellular sodium, reducing the concentration gradient of sodium which in turn decreases the activity of the sodium-calcium exchanger. As a result, less calcium is transported out of the cell leading to an increase in the concentration of intracellular calcium; triggering release of further calcium from the sarcoplasmic reticulum. The dose-dependent effect of intracellular calcium on contractility is well documented^33,34^ and is in part due to higher level of calcium binding to troponin C as it is one of the main regulators of contraction in cardiac muscle^35^. Digoxin does not affect the activation or relaxation of cardiac tissue, reflected in no effect being seen during the activation and passive re-lengthening phases of the cardiac work-loop (Fig. 3b). As detailed in Table 2, the concentration of digoxin used here was comparable to the concentration used in other *in vitro* contractility experiments^36–39^. Rosic *et al*.^38^ reported roughly a 25% increase in left-ventricular diastolic pressure following infusion of 5 µM Digoxin in Langendorff perfused guinea pig hearts. Guo *et al*.^39^ reported that 1 µM Digoxin caused a 31% increase in Ca^2+^ -wave amplitude in human induced pluripotent stem cell cardiomyocytes (hiPSC-CM)^39^.

Both dobutamine and isoprenaline are adrenoreceptor agonists; dobutamine is predominantly a β_1_ and α_1_ receptor agonist with weak β_2_ activity, isoprenaline activates both β_1_ and β_2_ receptors. Activation of β_1_-adrenoreceptors activates the G_S_ subunit of the adrenoceptor which initially directly activates the L-type calcium channel. Secondly, it activates adenylyl cyclase to form cAMP using ATP. An increase in the level of cAMP activates cAMP dependent protein kinase (protein kinase A) which phosphorylates numerous targets including: L-type calcium channels, phospholamban and troponin I^40^. Phosphorylation of L-type calcium channels results in a greater availability of intracellular calcium during systole which consequently increases contractility during the subsequent diastole^40^. This effect was observed in this study as an increase in force during shortening of the muscle. Phosphorylation of phospholamban causes more rapid removal of calcium from the cytosol into the sarcoplasmic reticulum enhancing relaxation of the muscle. Finally, enhanced calcium dissociation from myofilaments during diastole due to phosphorylation of troponin I which enhances relaxation of the muscle^40,41^. Increased levels of relaxation were also observed experimentally with lower forces being recorded during passive re-lengthening of the muscle. Layland *et al*.^28^ also observed an acceleration of twitch relaxation with 5 µM of isoprenaline. Brown and Erdmann^10^ observed ~60% increase in the isometric force in cat papillary muscles when perfused with ~50 µM dobutamine this is a similar amplitude of response as observed in this study at 1 µM.

Although both isoprenaline and dobutamine elicit their effects through similar mechanisms and their effects on the shape of the work-loop are similar, the magnitude and duration of the inotropic effect was much lower with isoprenaline. This may in part be due to the concentration of isoprenaline used. A dose response study was carried out for isoprenaline 10 nM–1 µM (data not shown here); it was found that concentrations of 100 nM and above caused the muscle to contract spontaneously as a result of its chronotropic and proarrhythmic properties. Hence, in this study a concentration of 30 nM was used. The addition of a chelating agent such as EDTA or EGTA would have inhibited the oxidation of isoprenaline^28^ and may have resulted in a greater increase in power output.

### Negative inotropes

Verapamil causes negative inotropy by blocking both L-type and T-type voltage-gated calcium channels^42^. This causes a decrease in the intracellular calcium levels which reduces the probability of the opening of the intracellular ryanodine receptors^43^. As a result, treatment with verapamil reduces both the probability of contraction and the force of contraction. Verapamil reduced the power output of the muscle during shortening and also reduced the force required to re-lengthen the muscle, it had no effect on the activation of the muscle (Fig. 6b) As shown in Table 2, the concentration used in this study is comparable to the concentration tested previously by Noguchi *et al*.^44^; a cumulative dose-response (1 nM–100 µM) for verapamil was carried out using guinea pig papillary muscles which underwent isometric contractions at frequency of 1 Hz. At a cumulative dose of 1 µM the developed force was ~40% compared to the control, however, the duration of each dose is unknown. In the current study the duration of drug treatment was restricted to 60 minutes meaning verapamil may not have elicited its maximal effect.

Flecainide is a Na_v_1.5 sodium channel blocker and an inhibitor of ryanodine receptor 2 (RyR2)^45^. RyR2 is a major regulator of the release of calcium from the sarcoplasmic reticulum and so inhibition results in a reduction in contraction. In this study, flecainide has been shown to be a negative inotrope it has been shown to exhibit its effects during the shortening and passive re-lengthening phases of the work-loop (Fig. 8b). A dose response for flecainide was also carried out 0.3 µM–10 µM (data not shown here), whereby a small but significant negative effect was observed at 0.3 µM which is comparable to the clinical concentration in Table 2. In an isometric contraction study^46^ flecainide was tested using rabbit papillary muscles. Josephson *et al*. observed a decrease in developed force of around 20% at a concentration of 5 µg/ml (~12 µM) and a significant decrease (p < 0.005) of around 30% at a concentration of 10 µg/ml (~24 µM). The magnitude of the response is comparable to this study, however, the concentrations used were significantly higher. Flecainide was tested in a cumulative concentration-response study using human trabecular bundles^47^; 3 µM flecainide caused ~15% reduction in peak isometric force compared to vehicle. This response was not significant (p > 0.05) however the magnitude of the response is comparable to this study.

In this study, atenolol has been shown to be a negative inotrope. Atenolol is a β_1_-adrenergic receptor blocker; it blocks endogenous adrenaline and noradrenaline. It has been demonstrated that electrical stimulation of isolated cardiac muscle elicits a contraction accompanied by noradrenaline release^48,49^. It has also been demonstrated that this adrenergic response to field stimulation can be markedly reduced by propranolol (a β_1_ and β_2_ adrenergic receptor blocker). Therefore, atenolol may also significantly reduce noradrenaline release and hence reduce contractility^50^. Atenolol also acts as an inverse agonist on β_1_ and β_2_ adrenoceptors, blocking receptors which are constitutively active^9,51^. It is hypothesised that the observed response was due to atenolol blocking endogenous adrenaline and noradrenaline and also acting as an inverse agonist. Atenolol exhibited its effects during the shortening and passive re-lengthening phases of the work-loop. Varma *et al*.^9^ tested atenolol at various concentrations in the right atria, left atria, right ventricles and isolated papillary muscles from the rat myocardium. They carried out isometric contractions at a frequency of 1 Hz. They found that there was no difference in contractile force when papillary muscles were treated with atenolol (10 nM–100 µM). A significant effect was measured in both the right and left atria at concentrations of above 100 nM. Kerns *et al*.^52^ tested atenolol using the rat Langendorff perfused heart model. Using a concentration of 25 mg/mL (~9.4 µM) atenolol significantly (p < 0.05) reduced left ventricular pressure after 60 minutes. Clinically, atenolol has been observed to cause a reduction in systolic and diastolic blood pressures of around 15% and a reduction in cardiac output of around 20%^53^ which is of similar magnitude to the reduction in power that has been observed in this study.

In the present study, the work-loop technique incorporated an electrical stimulation along with a sinusoidal length change which mimics the cardiac cycle *in vivo* more closely than other contractility techniques. Incorporating a physiologically relevant length change is one of the main advantages of the work-loop technique over other *in vitro* techniques such as isometric contraction studies. This work has successfully shown that the cardiac work-loop technique is useful in assessing the effects of inotropic agents on cardiac muscle contractility since it is sensitive to changes in force, velocity, activation rate and frequency. Further assessment of the effects of: phosphodiesterase type 3 inhibitors (e.g. pimobendan or milrinone), calcium sensitizers (e.g. levosimendan), Na^2+^ channel blockers (e.g. disopyramide) and those with more complex mechanisms of action (e.g. amitriptyline, quinidine) would support additional validation of the work-loop assay in the future.

In conclusion we have demonstrated that the work-loop model provides a more physiologically realistic technique for studying isolated cardiac muscle. The work-loop model enables the mechanical work of the muscle to be assessed. This is the first study in which known inotropes of various classes have been tested in the cardiac work-loop at a physiologically relevant temperature in order to assess its suitability as a contractility assay. By correctly identifying the inotropes this technique has been shown to have the potential to be used as a preclinical assay for contractility. This type of work-loop model has the potential to enable researchers to understand mechanical defects underlying cardiac diseases and subsequently study drug effects in various disease conditions. This is the first study in which changes in work-loop shape detailing for example the activation, shortening or passive re-lengthening have been linked to the mechanism of action of the compound and is a useful method with which to assess detailed mechanisms of drug-induced effects on cardiac muscle contractility.
